# Total Number of Identified Parathyroid Glands During Total Thyroidectomy and Its Relation to Postoperative Hypoparathyroidism

**DOI:** 10.7759/cureus.50597

**Published:** 2023-12-15

**Authors:** Bushra A Alharbi, Latefa A Alareek, Saleh Aldhahri, Saleh Alqaryan, Mohammed Al Essa, Khalid Al-Qahtani

**Affiliations:** 1 Otolaryngology - Head and Neck Surgery, Department of Otolaryngology, Head and Neck Surgery, King Saud University Medical College, Riyadh, SAU

**Keywords:** and reimplantation, risk factors, hypoparathyroidism, parathyroid gland, thyroidectomy

## Abstract

Objectives: To evaluate the correlation between the intraoperative number of identified parathyroid glands (PGs) and the risk of developing hypoparathyroidism postoperatively. Also, to determine the risks and prognostic factors in patients with postoperative hypoparathyroidism.

Methods: A retrospective study of 499 patients who underwent total thyroidectomy at two tertiary care institutions, King Saud University Medical City (KSUMC) and King Fahad Medical City (KFMC) in Riyadh, Saudi Arabia was included. Individual demographic characteristics with detailed clinical information were recorded, focusing mainly on operative reports for a total number of identified PGs intraoperatively and investigating the risk of developing hypoparathyroidism postoperatively. Factors such as age, gender, comorbidity, and number of excised and reimplanted parathyroid glands were investigated to determine the risks and prognostic factors in patients with postoperative hypoparathyroidism.

Results: The findings from the analysis showed that the number of identified PGs intraoperatively had a positive correlation with a higher postoperative risk of developing hypoparathyroidism. For zero, one, two, three, and four identified PGs, the risk of hypoparathyroidism in one-hour parathyroid hormone level postoperative was 6.6%, 7.3%, 34.4%, 34.4%, and 17.2% respectively.

Conclusion: The greater the number of identified PGs intraoperatively, the less likely it was to prevent inadvertent hypoparathyroidism post-total thyroidectomy.

## Introduction

Total thyroidectomy is one of the most if not the most common surgeries performed especially by head and neck surgeons as a surgical option to treat various thyroid neoplasms. Furthermore, like many other surgical procedures, it can cause a variety of complications, one of which is postoperative hypoparathyroidism [1]. The prevalence of hypoparathyroidism after total thyroidectomy contrasts broadly based on the group of individuals studied, surgical methods, and definition used for explaining hypoparathyroidism [2]. According to previous research, transient hypoparathyroidism, which occurs after 20-70% of total thyroidectomies, resolves itself within six months and doesn't require calcium or vitamin D supplements. Moreover, permanent hypoparathyroidism denotes hypocalcemia after six months of surgery and indicates the requirement for calcium or vitamin D supplements to sustain normal calcium levels. It is reported that around 1-9% of cases require calcium supplements postoperatively [3], although the frequency has risen to 18% in recent years [4,5].

Regarding the direct cause of inadvertent post-thyroidectomy hypoparathyroidism, certain situations such as surgery for central neck dissection [3,5], malignancy [5], and unintentional parathyroidectomy [1,3,5] have been linked to it in the literature. Over time, it was primarily avoided by widespread consensus that, in order to prevent hypoparathyroidism following thyroid surgery, all four parathyroid glands (PGs) and their vascular supply must be preserved. However, because of their variable anatomic localization, during thyroid surgery, it is not usually possible to locate all four parathyroid glands [6,7]. Furthermore, a majority of authors agree that vigorous search and dissection pose a risk for devascularization [8-10]. A number of strategies have been put forth to maintain the function of parathyroid glands after surgery, but clinicians are still unsure about the best strategy to use when performing a total thyroidectomy in order to reduce the risk of hypoparathyroidism.

Therefore, our study aimed to assess the relationship between the risk of hypoparathyroidism following a total thyroidectomy procedure and the total number of identified PGs. Also, to determine the risks and prognostic factors among patients with postoperative hypoparathyroidism.

## Materials and methods

This retrospective study was conducted at two tertiary care institutions, namely King Saud University Medical City (KSUMC) and King Fahad Medical City (KFMC) in Riyadh, Saudi Arabia.

We pursued and successfully obtained ethical approval from the Institutional Review Board (IRB) at KSUMC and the IRB at KFMC. Approval for the study was obtained from the KSUMC and KFMC Institutional Review Boards on March 31st, 2022 (E-22-6785 and 22-398, respectively). Due to the retrospective nature of this paper, informed consent was waived.

Patients who underwent total thyroidectomy surgery between January 2010 to December 2022 were included in the study. The indications for total thyroidectomy included multinodular goiter, Graves' disease, suspicious thyroid nodules and malignant thyroid nodules. We excluded patients who underwent central neck dissection, hemithyroidectomy or surgery for other head and neck tumors.

The main emphasis was on the intraoperative assessment of the total number of PGs identified during the surgery and assessing the risk of postoperative hypoparathyroidism. Throughout the surgery, careful attention was given to the distinct features of the parathyroid glands, including their caramel color, firmness, and round shape. A parathyroid gland was considered positively identified only when it exhibited all the mentioned characteristics, and any glands presumed to be parathyroid were excluded from the count.

Using patient charts, we gathered data on medical record numbers and intraoperative reports (parathyroid gland identification, inadvertent excision, and re-implantation). A comprehensive chart review examined patient characteristics, demographics, parathyroid outcomes, and thyroid pathology findings. To identify risks and prognostic factors for postoperative hypoparathyroidism, we analyzed variables such as age, gender, comorbidities, and the number of identified parathyroid glands intraoperatively and in pathology reports.

Biochemical hypoparathyroidism is characterized by a parathyroid hormone (PTH) level below 1.7 mmol/L. The most reliable predictor of postoperative hypocalcemia is the mean relative decrease in PTH measured at one hour and six hours after surgery [10]. To assess this, patients were monitored for preoperative PTH levels at one hour, six hours, and discharge time. Additionally, preoperative levels of magnesium and vitamin D were also recorded.

For statistical analysis, continuous variables were summarized using mean and standard deviation, while median and inter-quartile range (IQR) were used for other continuous variables. Categorical variables were presented as frequencies and percentages. Correlations between intraoperative identified PGS and postoperative hypoparathyroidism risk were assessed using the chi-squared test. The multivariable binary logistic regression (MLBR) determined the significance of predictor variables in explaining the association with hypoparathyroidism risk at discharge, presented as odds ratios (OR) with a 95% confidence interval. Statistical analysis was performed using Statistical Package for Social Sciences (SPSS) version 21 (IBM Corp., Armonk, NY, USA), and significance was set at p < 0.05.

## Results

A total of 449 post-thyroidectomy patients’ medical records were collected and reviewed, of which 78.4% were females and 21.6% were males. Around 48.3% of patients were observed to harbor one or more than one comorbidity. The rest of the yielded descriptive analysis findings for the sample characteristics are shown in Table 1.

**Table 1:** Descriptive data of the patients' sample characteristics *Quantities are counts: (gender, number of parathyroid glands (PGs) identified, number of PGs found in pathology specimen, Bethesda category)

Descriptive data	Quantization* N=449
Female gender	352 (78.4%)
Male gender	97 (21.6%)
No PGs identified	36 (8%)
One PG identified	83 (18.5%)
Two PGs identified	162 (36.1%)
Three PGs identified	116 (25.8%)
Four PGs identified	52 (11.6%)
No PGs found in the pathology specimen	359 (80%)
One PG found in the pathology specimen	79 (17.6%)
Two PGs found in the pathology specimen	8 (1.8%)
Three PGs found in the pathology specimen	3 (0.7%)
Four PGs found in the pathology specimen	0 (0%)
Bethesda category I	13 (3.2%)
Bethesda category II	152 (37.4%)
Bethesda category III	100 (24.6%)
Bethesda category IV	41 (10.1%)
Bethesda category V	10 (2.5%)
Bethesda category VI	91 (22.4%)

Intraoperatively, the number of identified PGs during total thyroidectomy is recorded as follows: 8% of patients had no parathyroid gland identified, one, two, three, and four parathyroid glands were identified in 18.5%, 36.1%, 25.8%, and 11.6% of patients respectively. Furthermore, histopathological investigation of thyroid glands revealed that 80% of patient samples contained no parathyroid glands, one, two, and three parathyroid glands were found in 17.6%, 1.8%, and 0.7% respectively in patient samples. Moreover, 8% of patients underwent intraoperative reimplantation of parathyroid glands.

The study also assessed serum magnesium, serum parathyroid hormone, and vitamin D levels, providing insights into their distribution and changes over time (Table 2). There was a slight decrease in pre- and postoperative serum Mg levels (0.80 and 0.75 respectively) and the serum vitamin D level was 55.31 ng/ml.

**Table 2:** Descriptive analysis of measured serum electrolytes, parathyroid hormone level and parathyroid glands function outcomes. *Quantities are counts, mean ± SD (PTH, Mg, and vitamin D serum levels), or percent (normal or low PTH level). Mg: magnesium, PTH: parathyroid hormone, SD: standard deviation

Laboratory tests	Quantization* N=449
Preoperative serum Mg Level (mmol/L), mean ± SD	0.80 ± 0.10
Postoperative serum Mg Level (mmol/L), mean ± SD	0.75 ± 0.11
Preoperative serum PTH Level (pg/ml), mean ± SD	7.33 ± 5.81
Preoperative serum vitamin D Level (ng/ml), mean (SD)	55.31 ± 29.75
Preoperative normal PTH level	399 (88.9%)
Preoperative low PTH level	50 (11.1%)
Postoperative serum PTH Level at 1 hour (pg/ml), mean ± SD	3.67 ± 2.97
Postoperative normal PTH level at 1 hour	298 (66.4%)
Postoperative low PTH level at 1 hour	151 (33.6%)
Postoperative serum PTH Level at 6 hours (pg/ml), mean (SD)	3.71 ± 2.93
Postoperative normal PTH level at 6 hours	305 (67.9%)
Postoperative low PTH level in 6 hours	144 (32.1%)
Discharge time serum PTH level (pg/ml), mean ± SD	2.93 ± 1.29
Discharge time normal PTH level	328 (73.1%)
Discharge time low PTH level	121 (26.9%)

For estimating the correlation between number of identified PGs intraoperatively and postoperative risk of hypoparathyroidism, a bivariate chi-square test of independence was performed. It was observed that as the number of identified PGs tends to increase, the patient’s risk of postoperative hypoparathyroidism tends to rise accordingly. Intraoperative number of identified PGs was zero, one, two, three, and four and the risk of hypoparathyroidism in one hour PTH level was 6.6%, 7.3%, 34.4%, 34.4%, and 17.2% respectively. When two or more parathyroid glands are found intraoperatively, parathyroid hormone levels are seen to rise and then stabilize throughout the course of the evaluation time points (Table 3). 

**Table 3:** The correlation between the number of intraoperatively identified parathyroid glands with the risk of hypoparathyroidism post-surgery. PTH: parathyroid hormone, PGs: parathyroid glands. * p-value for the chi-squared test of association.

Number of identified PGs	One-hour postoperative PTH level	Six-hour postoperative PTH level	Discharge time postoperative PTH level
None	10 (6.6%)	9 (6.2%)	7 (5.8%)
One	11 (7.3%)	12 (8.3%)	11 (9.1%)
Two	52 (34.4%)	52 (36.1%)	42 (34.7%)
Three	52 (34.4%)	50 (34.7%)	42 (34.7%)
Four	26 (17.2%)	21 (14.6%)	19 (15.7%)
*p-value	<0.001	<0.001	0.002

On multivariate logistic binary regression analysis, significant correlations were observed between distinct factors and the probability of experiencing post-thyroidectomy hypoparathyroidism. Gender played a role, with males being 1.529 times more likely to develop hypoparathyroidism in comparison to females (p=0.032). The patient’s age showed a significant negative correlation, indicating that for each additional year, the chances of experiencing hypoparathyroidism decreased by 1.9% (p=0.007). A pre-surgical history of hyperthyroidism significantly increased the probability; a positive history is associated with 3.126 times higher chances of developing hypoparathyroidism (p=0.002). The total number of parathyroid glands inadvertently removed during surgical treatment was positively correlated, with each additional gland removed increasing the probability by 14.1% (p<0.001). 

Longer hospital stays were associated with a higher probability of hypoparathyroidism (p<0.001). Pre-surgical serum magnesium levels showed a significant negative correlation, indicating that for each additional mg/dL, the probability of post-thyroidectomy hypoparathyroidism decreased by 94.1% (p=0.016). Conversely, the pre-thyroidectomy serum vitamin D levels had a significant positive correlation, with each additional ng/ml increasing the probability of lower parathyroid hormone levels by 0.5%. Furthermore, patients who underwent re-implantation following inadvertent surgical removal of the parathyroid gland were found to be significantly more likely (1.687 times more) to experience post-surgical low serum PTH levels from the time of admission to discharge as compared to the patients who did not undergo parathyroid gland re-implantation (p=0.029).

## Discussion

The determination of the total count of parathyroid glands during total thyroidectomy surgery is a subject of debate in the literature regarding its potential impact on the occurrence of postoperative hypoparathyroidism. The conclusive answer to this question remains elusive due to the varied findings and relatively low incidence in prior studies [1]. Our investigation aimed to ascertain the intraoperative identification of PGs and the corresponding percentages associated with hypoparathyroidism development. Our findings indicate that an increase in the number of identified PGs corresponds to a higher risk of postoperative hypoparathyroidism. Specifically, when none, one, two, three, and four PGs were identified intraoperatively, the risks of postoperative hypoparathyroidism were 6.6%, 7.3%, 34.4%, 34.4%, and 17.2%, respectively.

A study by Riordan et al. aligned with our observations, revealing that patients with fewer preserved PGs experienced a lower incidence of postoperative biochemical hypocalcemia and hypoparathyroidism compared to those with a greater number identified [11]. Similarly, Prazenica et al. reported an increased incidence of postoperative hypoparathyroidism in cases with a higher number of identified PGs [12].

In our assessment of the number of identified PGs, patients with one or no identified parathyroid glands exhibited the lowest risk of developing hypoparathyroidism. This raises the question of whether dissecting parathyroid glands serves as an indicator of increased hypoparathyroidism risk, potentially advocating against routine identification [2]. Contrary findings by Grimm et al. suggested a significant reduction in permanent hypoparathyroidism if all four parathyroid glands were not visualized during thyroidectomy [13,14]. Nonetheless, some research supports the traditional recommendation of identifying all four glands to minimize postoperative hypoparathyroidism risk [15,16]. Olson et al. emphasized that preserving less than three glands significantly increases the risk of hypoparathyroidism [17].

However, Sitges-Serra et al.'s report demonstrated an inverse relationship between the total number of parathyroid glands determined and the occurrence of permanent deficiency [5]. Conflictingly, some researchers found no correlation between identifying a higher number of parathyroid glands and reduced postoperative hypoparathyroidism risk; in fact, they observed the opposite [3,9,13].

This discrepancy is underscored by studies questioning the systemic identification of all four parathyroid glands, suggesting that meticulous dissection may compromise blood supply, especially if it involves hemostasis methods that can harm the glands [7]. Despite their extracapsular location, the variability in parathyroid gland positions complicates systemic identification in every case [17].

Inadvertently detached parathyroid glands during thyroid surgery can be reimplanted as a recommended surgical treatment [18,19]. In our study, 8% of patients underwent intraoperative reimplantation, indicating a negative impact on postoperative parathyroid gland function. Patients who underwent auto-transplantation were 1.687 times more likely to experience postoperative hypoparathyroidism compared to those without reimplantation. Similar findings by Gschwandtner et al. and Lo et al. supported the notion that reimplantation negatively affects postoperative parathyroid function [3,20]. Conversely, Tartaglia et al. found no effect on hypocalcemia or the frequency of permanent hypoparathyroidism with reimplantation [21].

Preoperative vitamin D levels were identified as a significant risk factor for postoperative hypoparathyroidism. Our retrospective study showed a positive correlation between preoperative serum vitamin D levels and the probability of lower parathyroid hormone levels. In contrast, Martins et al. reported a negative correlation between PTH and vitamin D levels [22], while Kilicarslan et al. found that over 75% of vitamin D-deficient patients had normal PTH levels [23].

Our study has limitations, primarily due to its retrospective nature and subjectivity in determining intraoperative parathyroid gland numbers. Secondly, we did not take into consideration single versus multiple surgeons and the experience of the surgeons. However, the present study was implemented within two tertiary healthcare centers. This multi-center approach strives to mitigate potential biases associated with a single-center study and offers insights that are more representative of diverse clinical settings.

## Conclusions

Number of identified PGs intraoperatively does not correlate with the prevention of hypoparathyroidism post-total thyroidectomy. Patients with one or no identified parathyroid glands had the lowest risk of developing hypoparathyroidism. In addition, it was noticed that patients who underwent reimplantation were at a higher risk of postoperative hypoparathyroidism compared to those without reimplantation. These findings imply that a selective approach where parathyroid glands are encountered during a total thyroidectomy is not inferior but rather preferable to actively seeking out for identification of all four parathyroid glands in every case.
